# Supplementary material for: Causal interplay between lactose intolerance and gut microbiota: a combined bidirectional Mendelian randomization and in vivo validation study
Source: Front Nutr. 2026 Jun 1;13:1803337. doi: 10.3389/fnut.2026.1803337 (PMC13265576; doi:10.3389/fnut.2026.1803337)
Supplement: Supplementary file 1 [file Data_Sheet_1.zip › supplementary materials/Forward/leave one out cross validation/ebi-a-GCST90027547.finngen_R12_E4_LACTONAS.pdf]

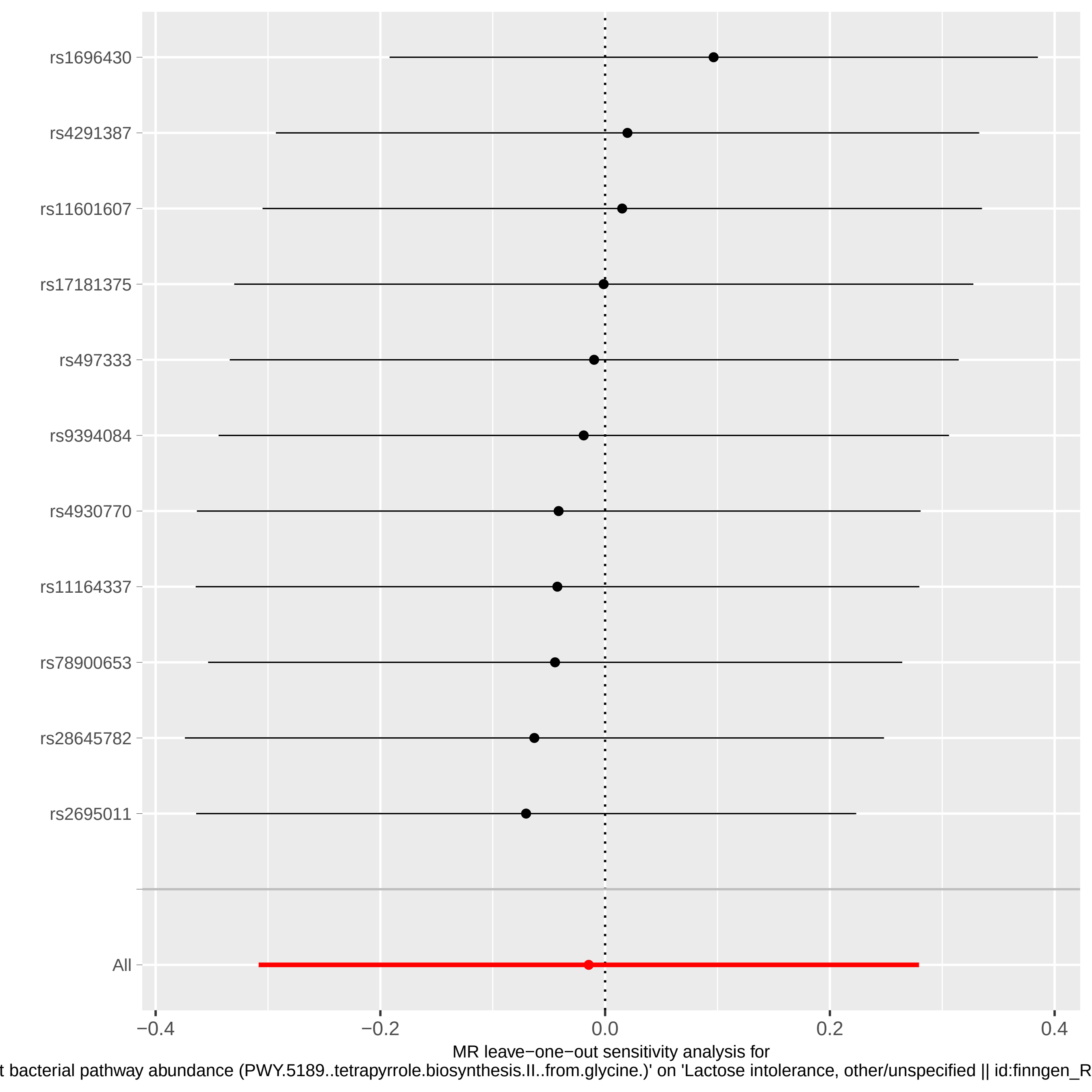

rs1696430
rs4291387
rs11601607
rs17181375
rs497333
rs9394084
rs4930770
rs11164337
rs78900653
rs28645782
rs2695011
All
−0.4
−0.2
0.0
0.2
0.4
MR leave−one−out sensitivity analysis for
t bacterial pathway abundance (PWY.5189..tetrapyrrole.biosynthesis.II..from.glycine.)' on 'Lactose intolerance, other/unspecified || id:finngen_R
